# Controllable vacuum-induced diffraction of matter-wave superradiance using an all-optical dispersive cavity

**DOI:** 10.1038/srep35402

**Published:** 2016-10-17

**Authors:** Shih-Wei Su, Zhen-Kai Lu, Shih-Chuan Gou, Wen-Te Liao

**Affiliations:** 1 Department of Physics and Graduate Institute of Photonics, National Changhua University of Education, Changhua 50058, Taiwan; 2 Max Planck Institute for Quantum Optics, D-85748 Garching, Germany; 3 Department of Physics, National Central University, 32001 Taoyuan City, Taiwan; 4 Max Planck Institute for the Structure and Dynamics of Matter, 22761 Hamburg, Germany; 5 Max Planck Institute for the Physics of Complex Systems, 01187 Dresden, Germany; 6 Center for Free-Electron Laser Science, 22761 Hamburg, Germany

## Abstract

Cavity quantum electrodynamics (CQED) has played a central role in demonstrating the fundamental principles of the quantum world, and in particular those of atom-light interactions. Developing fast, dynamical and non-mechanical control over a CQED system is particularly desirable for controlling atomic dynamics and building future quantum networks at high speed. However conventional mirrors do not allow for such flexible and fast controls over their coupling to intracavity atoms mediated by photons. Here we theoretically investigate a novel all-optical CQED system composed of a binary Bose-Einstein condensate (BEC) sandwiched by two atomic ensembles. The highly tunable atomic dispersion of the CQED system enables the medium to act as a versatile, all-optically controlled atomic mirror that can be employed to manipulate the vacuum-induced diffraction of matter-wave superradiance. Our study illustrates a innovative all-optical element of atomtroics and sheds new light on controlling light-matter interactions.

When atoms and photons are confined in a cavity, they behave differently from what they do in free space. Owing to its simplicity, cavity quantum electrodynamics (CQED)^1^ has facilitated the realization of quantum technology, including quantum metrology^2^, quantum teleportation^3^, quantum logic gates^4^, quantum memory^5^ and so forth. Given the above feats, the ability to readily build a scalable quantum logic network composed of interacting CQED nodes is expected. This, however, is nontrivial when controllability and strong coupling are both required. Here, we investigate a CQED system consisting of a binary Bose-Einstein condensate (BEC)^6^,^7^,^8^,^9^,^10^ sandwiched in between two separated atomic clouds in the presence of electromagnetically induced transparency (EIT) mechanism^11^,^12^, namely, a dispersive cavity or an EIT-based cavity. The highly tunable EIT dispersion enables an all-optical control over cavity mirrors^11^,^12^,^13^,^14^,^15^,^16^ and therefore over BEC-cavity field interactions. Apart from its controllability, the mode volume of the EIT-based cavity can be finely adjusted by changing trapping potentials of atomic mirrors, and so the cavity spacing can be comparable to that of a conventional one. However, in essence, the present scheme does not share the same technical problems with conventional cavity when pushing the limit to a smaller mode volume. On the other hand, instead of a dynamically controllable object, superradiance results from quantum fluctuation^17^ and has long-term been considered as a consequence of the geometry or concentration of its emitters^18^, e.g., Bose-Einstein condensate (BEC)^19^. Here, by taking advantage of the controllability of the EIT-based cavity, we demonstrate the possibility of controlling over both superradiance and matter waves using our system. We show that tight confinement, and nonlinear nature of BEC, lead to vacuum-induced diffraction of matter-wave superradiance^7^,^8^,^20^. This effect has never been addressed and is the signature of the strong coupling region where our CQED system is functional. In view of these advantages, without the restriction of conventional mirrors, our CQED system can be integrated on atom chips^21^, due to its tiny size, and it can also be used to study the cavity-assisted spin-orbit coupling in ultracold atoms^22^, bringing both dynamical controllability and miniaturization into full play and providing a non-mechanical control over CQED.

As depicted in Fig. 1(a), the dispersive cavity consists of two separate ensembles of 3-level atoms of Λ type. Based on EIT^13^,^23^,^24^,^25^,^26^, as illustrated in Fig. 1(b,d), each 3-level atomic medium constitutes a mirror when interacting with two counter-propagating coupling fields with Rabi frequencies 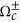 driving the 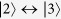 transition. As a consequence of four-wave mixing^26^, each EIT medium acts as a phase conjugate mirror^27^, where an incident probe field Ω^+^ driving the transition 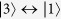 will be reflected and generate a backward probe field Ω^−^ 13,24,26, and vise versa. To demonstrate the controllability and novelty of the dispersive cavity, we consider a quasi one-dimensional (1D) binary BEC, which transversely flies through it and emits photons^8^. As illustrated in Fig. 1(c), a binary BEC consists of atoms condensed in the electronic states 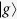 and 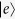, and the transition 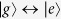 can be identical to 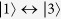. In general, when the selection rule and frequency of transition 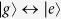 match that of 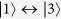 of two atomic mirrors, the emitted photons with wavelength *λ* from the BEC will be reflected by the EIT medium at either side. Quantum fluctuations^17^ and the geometry of the BEC will engender the spontaneous emission of supperradiance (SR) along the BEC’s long axis^17^,^18^,^19^. Thus, when the loaded BEC and the cavity are coaxial, the SR will travel back and forth inside the dispersive cavity and successively scatter with the condensate before they become subsequently absorbed by 3-level atoms when switching off the coupling fields. As demonstrated in the bottom part of Fig. 1(a), the interaction between cavity photons and the BEC renders the generation of matter-waves with high order harmonics of photon wave-number possible, namely, the diffraction of matter-waves^28^ following the release of a BEC from a trap. Because this effect is mostly related to vacuum fluctuations, we refer to it as vacuum-induced diffraction of matter-waves.

## Results

### The Model

The optical properties of the dispersive cavity are determined by the coupled Maxwell-Bloch equation^13^,^14^,^24^,^25^,^26^,^29^,^30^


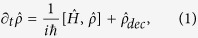



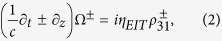


where 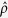 is the density matrix for the state vector 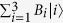 of the 3-level atom; 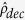 describes the spontaneous decay of the excited state |3〉 characterized by rate Γ^29^,^31^,^32^,^33^,^34^. 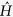 is the Hamiltonian describing the interaction between atomic mirrors and counter-propagating fields. In the presence of the counter-propagating coupling and SR fields, the total Hamiltonian of the EIT medium is given by 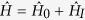 where 
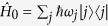
 and 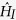 describes the atom-light interaction:


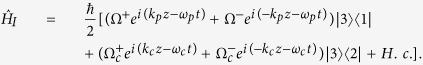


**Figure 2:**
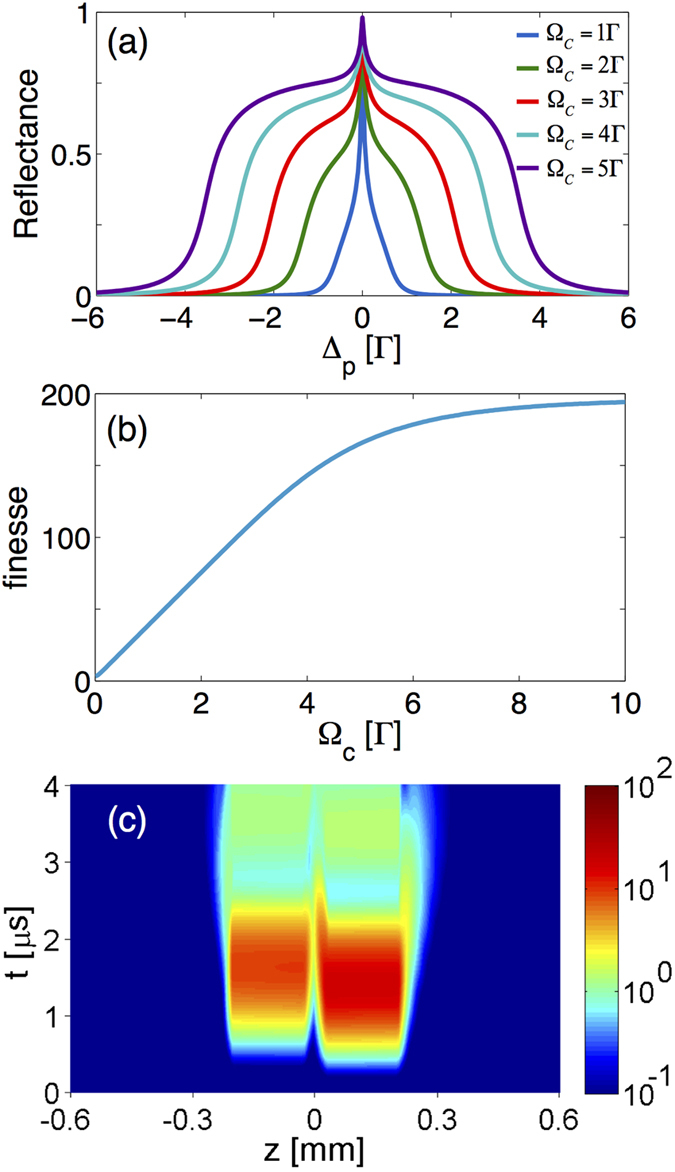
The reflectance of the atomic mirror and the enhancement of cavity finesse are shown in (**a**,**b**), respectively. Panel (**c**) shows the confined superradiant field emitted by the BEC, [|Ω^+^ (*t*, *z*)|^2^ + |Ω^−^(*t*, *z*)|^2^]/Γ^2^ with (*N*_*BEC*_, *A*) = (3 × 10^5^, 9*πμm*^2^), entrapped in the region of −0.2 mm < *z* < 0.2 mm.

*η*_*EIT*_ = Γ*d*^*opt*^/2*L* with *d*^*opt*^ the optical depth and *L* the length of the medium. Essentially, the reflection can take place at different positions in our model such that the penetrating depth of the cavity fields into each mirror medium is determined by Eqs (1) and (2). The dispersive cavity has single reflecting band whose width is associated with the EIT transparency window Δ*ω*_*EIT*_ ≅ Ω_*c*_ which is highly tunable by varying the coupling fields. As shown in Fig. 2(a), at various values of detuning and a fixed optical depth *d*^*opt*^ = 500, the reflectance approaches unity when the fields Ω^±^ are resonant and the width can be controlled by varying the intensity of the coupling fields. To gain more insight into the dispersive cavity, we invoke the effective cavity finesse *f*(Δ_*p*_) = *π*/[1 − *R*(Δ_*p*_)] where *R*(Δ_*p*_) is the reflectance as a function of the detuning Δ_*p*_ of fields Ω^±^ 35. The effective cavity finesse is plotted in Fig. 2(b), which demonstrates that the cavity finesse can be controlled by tuning the width of EIT transparency window^11^,^12^. The recoil of the EIT clouds causes Doppler shift 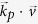 and acts as an effective detuning, where 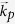 is the wave vector of the cavity field and 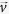 the velocity of an interacting atom. Such Doppler shift is around the order of 10^−4^Γ in typical ultracold atom experiments and therefore much smaller than both the nature line width Γ of atoms and the width of the EIT transparency window 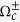. Technically, one can use external optical trap to confine the atomic ensembles, and so the recoil will not affect the position of the clouds. Therefore, in the present system, the recoil of the EIT clouds is negligible^24^,^25^. Moreover, without any rotation of the quantum axis, the polarizations of the incident and reflected cavity fields are identical due to the coherent EIT mechanism. Thus, the cavity field will couple to the same BEC transition in both directions. The full intra-cavity dynamics is described by Eqs (1) and (2) together with the coupled Gross-Pitaevskii equation for the BEC which reads


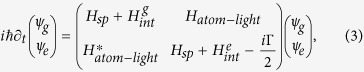


where (*ψ*_*g*_, *ψ*_*e*_)^*T*^ is the spinor wave function of binary BEC. The excited level |*e*〉 suffers from a spontaneous decay at a rate Γ, which decoheres the BEC wave function. The random nature of spontaneous emission plays the major role of the heating of condensed atoms which leads to the non-conservation of the BEC population^19^ (see Supplementary Information). In Eq. (3), the Hamiltonians involved in the coupled Gross-Pitaevskii equations are explicitly expressed as 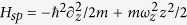, 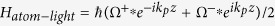 and 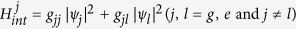 which are the single-particle, atom-light interaction, and nonlinear interaction Hamiltonians, respectively. The quasi-1D nonlinear interaction constant takes the form as *g*_*jl*_ = 2*πa*_*jl*_*ω*_*r*_*N*_*bec*_/*ω*_*z*_*a*_*osc*_ where 
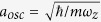
 is the oscillator length. In the presence of confined SR fields, the macroscopic wave functions and coherence contain contributions from the high ±*nk*_*p*_ modes due to the atom-light interaction and thus we can write 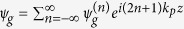 and 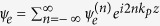 where 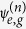 are both the spatially slowly-varying envelope. Therefore the optical coherence of the condensate, 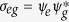
25, can be expanded as 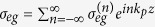, while the coherence terms, 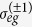, carrying 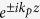 account for the generation of SR fields.

The initial fluctuations activating the supperradiant process are formulated by truncated Wigner method^36^. We assume that the BEC is initially prepared in a polarized state where only the electronic excited level |*e*〉 is macroscopically occupied. On the other hand, the electronic ground level |*g*〉 is scarcely occupied but is subjected to quantum fluctuations (see Methods). The dynamics of the generated SR fields is described by Eq. (2) except that 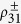 and *η*_*EIT*_ are replaced by 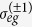 and *η*_*BEC*_, respectively. Here, 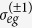 is the coherence of the BEC associated with the plane-wave 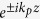 and *η*_*BEC*_ = 3Γ*N*_*BEC*_*λ*^2^/4*πA* with *A* being the transverse cross section of the BEC. We investigate the dynamics of SR fields and BEC by numerically integrate Eqs (1, 2, 3) (see Methods).

### Trapping SR Field in Atomic Cavity

In the following, we consider the experimental realization of an atomic mirror with a medium of optical depth *d*^*opt*^ = 500 and a coupling field of Ω_*c*_ = 2Γ. To this end, a quasi-1D BEC of ^7^Li at 2S_1/2_ (|*g*〉) state of *N*_*BEC*_ = 10^6^ can be prepared in a single beam dipole trap with the longitudinal and transverse trapping frequencies (*ω*_*z*_, *ω*_*r*_) = 2*π* × (100, 2.1 × 10^3^) Hz. An additional TEM_01_-type laser mode beam is applied to cross the atomic ensemble^37^. The spatial intensity distribution of the crossing beam allows to entrap low density thermal clouds aside the atomic mirrors while leaving the central BEC undisturbed. The narrow linewidth of 3P_3/2_ (|*e*〉) state, Γ = 760 kHz^38^ not only allows the coherent transferring of the ^7^Li BEC to the excited state by a *π* pulse, whose duration is shorter than 1/Γ, but also offers a technically feasible time scale for detection (see Supplementary Information for a study of *π* pulse pumping and the resulted heating effect in BEC). Moreover, *d*^*opt*^ = 1000 has already been experimentally achieved in cold atom systems^39^,^40^. As an example of a single numerical realization, the intensity of the supperradiant fields, |Ω^+^ (*t*, *z*)|^2^ + |Ω^−^(*t*, *z*)|^2^, emitted by the BEC is confined in a dispersive cavity as depicted in Fig. 2(c). The 3-level atomic mirrors are formed in the two spatial regions, 0.2 mm ≤ |*z*| ≤ 0.6 mm, and in between these two mirrors a quasi 1D BEC with a longitudinal Thomas-Fermi radius of 0.17 mm is harmonically trapped in the central domain, |*z*| < 0.2 mm. A flat cavity wall can be achieved by using a uniform box potential^41^. Thus, there is no overlap between three clouds. The detailed behavior of trapped SR fields changes from one realization to another depending on random initial fluctuations.

### Vacuum-Induced Diffraction

**Figure 3:**
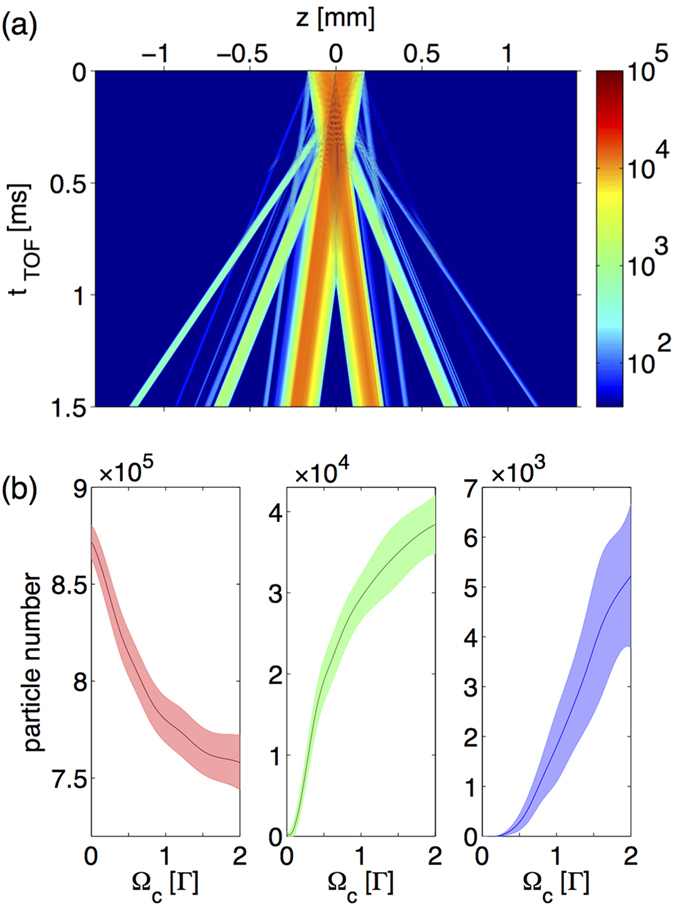
(**a**) The BEC’s TOF dynamics. Six flying lumps of atoms carrying momenta of ±*k*_*p*_, ±3*k*_*p*_, and ±5*k*_*p*_ can be observed at the end of TOF. (**b**) The coupling-field-strength-dependent mean population of different modes of the diffracted BEC carrying ±*k*_*p*_ (red), ±3*k*_*p*_ (green) and ±5*k*_*p*_ (blue). All the data points are averaged over 1000 realizations, and the shaded regions illustrate the corresponding error bars. Increasing the strength of the coupling field, the particle number of ±*k*_*p*_ mode decreases while the particle numbers of ±3*k*_*p*_ and ±5*k*_*p*_ modes increase, which demonstrates the coherent generation of high modes.

**Figure 4:**
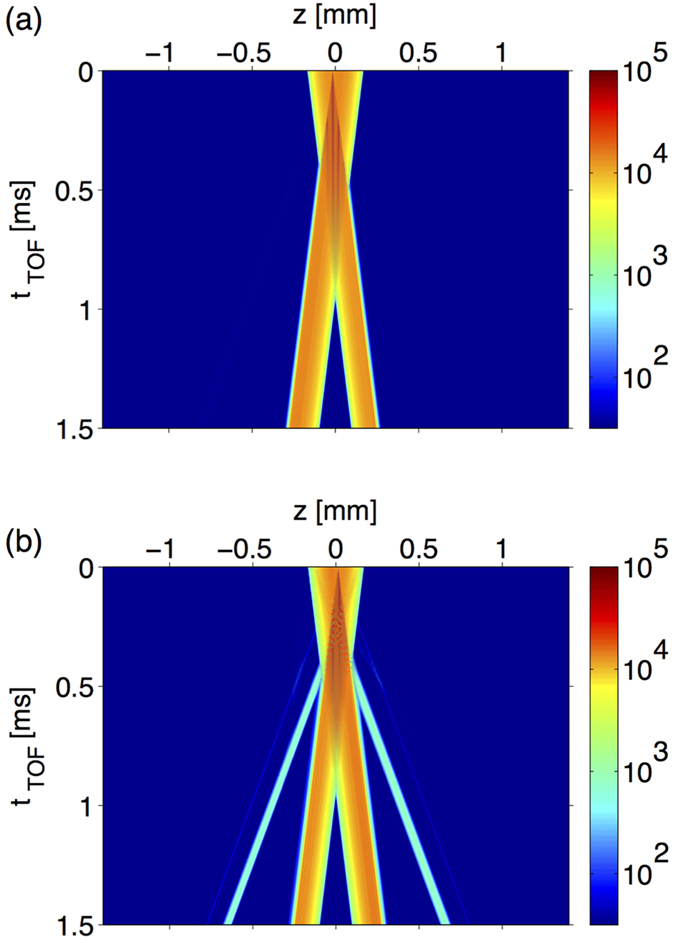
The dynamical evolution of the BEC after being released from the dispersive cavity is shown. In panel (a), the BEC is initially loaded into the cavity with the coupling field turned off at t = 0.02 *μ*s. In this case, only ±*k*_*p*_ modes are significantly generated with the corresponding particle numbers of each mode given by 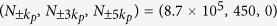. Panel (b) shows the TOF evolution of the BEC after interacts with the dispersive cavity with the coupling field on for 0.07 *μ*s. The longer interaction time enhances the coherent transfer of high *k*_*p*_ modes with the particle numbers of each mode 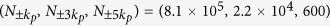.

The occurrence of vacuum-induced diffraction of matter-waves is prescribed in the operational sequence, namely, loading BEC into a dispersive cavity, promoting BEC to state |*e*〉 and then releasing BEC. Alternatively, one can also perform Raman scattering on BEC in a dispersive cavity. The vacuum fluctuations trigger the superradiant process that BEC emits SR photons and transit to the state |*g*〉. The emitted SR photons are confined in the dispersive cavity and interact with the BEC. Furthermore the atom-light interaction is enhanced due to the presence of cavity that the atoms experience the vacuum-induced Rabi oscillation (see Supplementary Information for the population dynamics). The Rabi oscillation results in the multiple scattering process that the condensed atoms are demoted and repumped between states |*e*〉 and |*g*〉. Therefore the strong coupling between cavity photons and BEC generates higher order harmonics (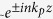) of matter waves in a dispersive cavity. These high *k*_*p*_ modes are disclosed by time-of-flight (TOF) simulation after the BEC is released from the cavity as shown in Fig. 3(a). Especially at t_TOF_ = 1.5 ms there are six flying lumps of atoms corresponding to −5*k*_*p*_, −3*k*_*p*_, −*k*_*p*_, *k*_*p*_, 3*k*_*p*_ and 5*k*_*p*_ (starting left). To characterize the process of generating high-*k*_*p*_ modes, we tune the cavity finesse by varying the strength of coupling fields and perform 1000 independent realizations for statistics. In Fig. 3(b), the population of ±*k*_*p*_ modes is gradually and coherently transferred to that of higher order harmonics with increasing Ω_*c*_. In contrast to the case of free space where only ±*k*_*p*_ modes and very small fraction of ±3*k*_*p*_ modes are generated, here the ±3*k*_*p*_ and ±5*k*_*p*_ modes are significantly populated when Ω_*c*_ ≳ 0.1Γ and Ω_*c*_ ≳ 1Γ, respectively. Note that the ±3*k*_*p*_ modes are enhanced by a factor of 10^3^. Additionally, the controllability of the dispersive cavity also provides the possibility to coherently manipulate the generation of BEC of high *k*_*p*_ modes. Such a protocol of dynamically manipulating the generation of matter-waves possessing a particular harmonic *k*_*p*_ can be realized by switching off two coupling fields at some instants to disable the dispersive cavity. By switching off the coupling fields, the SR fields is no longer confined but absorbed by the 3-level atomic mirrors, which also simultaneously turn off the coherent generation of high *k*_*p*_ matter waves. As shown in Fig. 4, we respectively turn off the coupling fields at 0.02 *μ*s and 0.07 *μ*s in Fig. 4(a,b) after BEC is loaded. After released form the cavity, the TOF evolution of the BEC shows a clear evidence that ±3*k*_*p*_ and ±5*k*_*p*_ modes can be successfully suppressed by releasing SR photons in due course before the corresponding multiple-scattering process occurs. We emphasize that the whole control procedure over both atomic mirrors and BEC remains coherent. While the absorption of each atomic mirror is coherently turned on, the interaction between BEC and the weakened cavity fields stays coherent. The present CQED system therefore demonstrates a dynamical controllability which is not available in conventional cavity systems.

## Discussion

In conclusion, we have studied the dynamics of a BEC interacting with SR in an all-optical dispersive cavity whose controllability has paved a way for the exquisite manipulation of light-mater interaction. Benefiting from the advances in ultracold atom experiments^42^,^43^,^44^, the spacing of the cavity can be finely adjusted by changing trapping potentials of atomic clouds. To estimate the mode volume, one can use 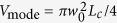 with *w*_0_ and *L*_*c*_ the beam waist and cavity spacing, respectively. We take *L*_*c*_ = 10 *μ*m and the beam waist is restricted by the transverse radius of the quasi 1D atomic mirrors which typically ranges from 1–10^2^ *μ*m giving that 2 *μm* ≤ *w*_0_ ≤ 200 *μm. V*_mode_ then ranges from 10^2^ *μ*m^3^ to 10^4^ *μ*m^3^, which makes strong light-atom coupling possible in our system. Whereas the present scheme utilizes the cooperative emission from BEC, a down-scaling of the system, e.g., the fundamental question like the behaviour of a single atom in a dispersive cavity deserves more detailed studies. As far as the applications in photonics are concerned, our system can serve as a prototype to create the entanglement of BEC and dark-state polaritons, or to be transformed into an all-optical Q-switching superradiant source^45^. Last but not least, recent advances in x-ray quantum optics^46^,^47^,^48^,^49^,^50^,^51^,^52^,^53^,^54^,^55^ suggest that atomic mirrors operated in an x-ray domain are realizable^56^, which suggests that a follow-up study of the current system incorporating x-ray quantum optics will be interesting and desirable.

## Methods

### Truncated Wigner method

The dynamics of the condensate flying through the cavity is simulated by the initial state where all the atoms are condensed in the excited state forming a Thomas-Fermi density profile while the ground state consists of quantum fluctuations sampled according to the truncated Wigner approximation of 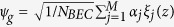, where *M* ≪ *N*_*BEC*_, *α*_*j*_ the random number satisfying 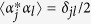, and all *ξ*_*j*_(*z*) form an orthonormal basis^36^.

### Numerical methods

Equations (1, 2, 3) are numerically solved by the method of lines where the Bloch and GP equations are propagated by the Fourier pseudospectral method and the adaptive Runge-Kutta method of orders 4 and 5 (RK45) for space and time integration, respectively, while the SR fields are integrated by the semi-Euler method. In the simulation, we use a large amount of Fourier modes, 5 × 2^12^, such that the cutoff momentum due to numerical discretization is high enough to describe the matter-wave scattering. Furthermore, the recoil-induced Doppler shift of different ±*nk*_*p*_ modes has been included intrinsically in our numerical calculation. In the truncated Wigner approximation, the number of mode for sampling initial fluctuations is *M* = 3000.

## Additional Information

**How to cite this article**: Su, S.-W. *et al.* Controllable vacuum-induced diffraction of matter-wave superradiance using an all-optical dispersive cavity. *Sci. Rep.* **6**, 35402; doi: 10.1038/srep35402 (2016).

## Supplementary Material

Supplementary Information

## Figures and Tables

**Figure 1:**
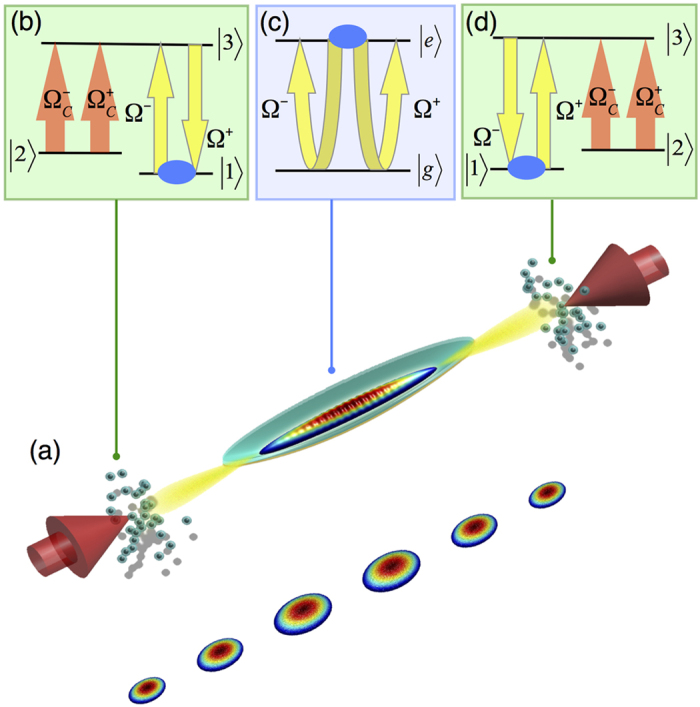
(**a**) A binary BEC initially condensed in |*e*〉 is sandwiched by two 3-level EIT media rendered by the transitions, 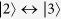 and 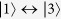, which are respectively driven by two counter-propagating coupling fields 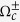 (red arrows) and probe fields Ω^±^ (yellow arrows) as shown in (**b**,**d**). The composition of 3-level atoms and coupling fields acts as a dispersive cavity. The bottom of panel (a) shows the splitting of the matter waves carrying different momenta during the time-of-flight expansion after released from the dispersive cavity. In (**c**), quantum fluctuations trigger the downward conversion of population from |*e*〉 to |*g*〉 in the binary BEC, activating the emission of forward and backward SR fields Ω^±^ which simultaneously serve as the probe fields.
