# Supplementary material for: Controllable vacuum-induced diffraction of matter-wave superradiance using an all-optical dispersive cavity
Source: Sci Rep. 2016 Oct 17;6:35402. doi: 10.1038/srep35402 (PMC5066314; doi:10.1038/srep35402)
Supplement: Supplementary Information [file srep35402-s1.pdf]

Supplementary Information: Controllable vacuum-induced diffraction of matter-wave superradiance using an all-optical dispersive cavity

Shih-Wei Su,[1] Zheng-Kai Lu,[2] Shih-Chuan Gou,[1,*] and Wen-Te Liao[3,4,5,6,†]

[1]Department of Physics and Graduate Institute of Photonics, National Changhua University of Education, Changhua 50058, Taiwan
[2]Max Planck Institute for Quantum Optics, D-85748 Garching, Germany
[3]Department of Physics, National Central University, 32001 Taoyuan City, Taiwan
[4]Max Planck Institute for the Structure and Dynamics of Matter, 22761 Hamburg, Germany
[5]Max Planck Institute for the Physics of Complex Systems, 01187 Dresden, Germany
[6]Center for Free-Electron Laser Science, 22761 Hamburg, Germany

In this supplementary material, we investigate the depletion of condensate during the π pulse pumping and present the equations of motion as well as the condensate dynamics in more details.

π pulse Pumping. The equations describing an optically pumped condensate read

$$i\hbar\partial_t\psi_g = \left(-\frac{\hbar^2}{2m}\partial_z^2 + \frac{m\omega_z^2 z^2}{2} + g_{gg}\left|\psi_g\right|^2 + g_{ge}\left|\psi_e\right|^2\right)\psi_g - \frac{\hbar\Omega_{pump}}{2}\psi_e,$$
$$i\hbar\partial\psi_e = \left(-\frac{\hbar^2}{2m}\partial_z^2 + \frac{m\omega_z^2 z^2}{2} + g_{ee}\left|\psi_e\right|^2 + g_{ge}\left|\psi_g\right|^2 - \frac{i}{2}\Gamma\right)\psi_e - \frac{\hbar\Omega_{pump}}{2}\psi_g, \quad \text{(S1)}$$

where Ω_{pump} is the Rabi frequency of the pumping beam and Γ is the spontaneous decay rate of the exited level $|e\rangle$, which decoheres the BEC wave function and plays the major role of heating mechanism. To investigate condensate depletion during the optical pumping in the presence of incoherent processes, we first prepare a initial state where all the atoms are condensed in the state $|g\rangle$ with the initial total particle number $N_{BEC} = 10^6$ and then turn on the pumping field with the duration Δt such that $\Omega_{pump}\Delta t = \pi$. In the numerical simulations, we consider four different pumping durations $\Delta t = 0.001\mu s$, $0.1\mu s$, $1\mu s$, and $10\mu s$. For the pulse duration shorter than $1/\Gamma \sim 1\mu s$ the condensed atoms in $|g\rangle$ can be coherently transferred to the excited level $|e\rangle$ as shown in Fig. S1. For the pulse duration shorter

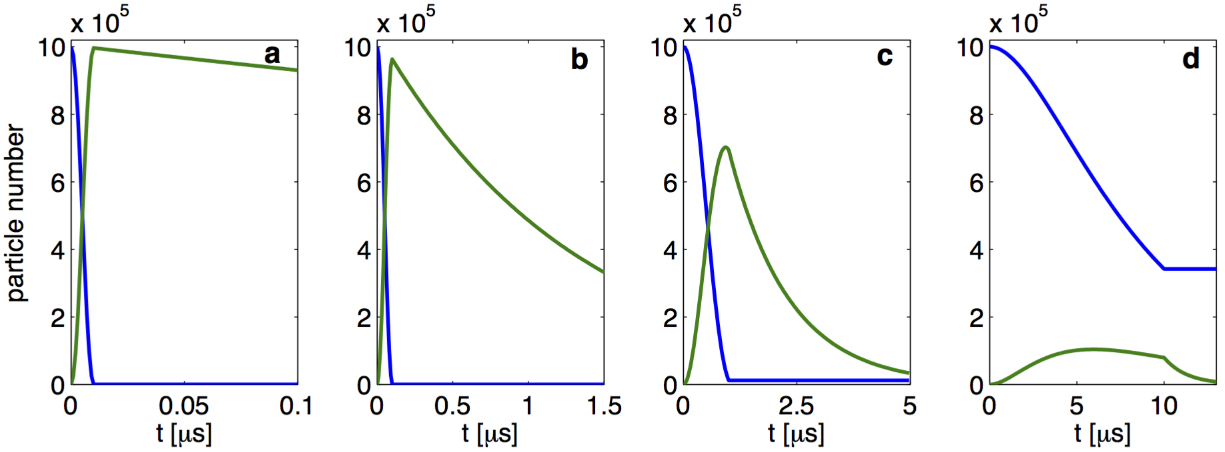

FIG. S1. Panels **a-d** show the condensate populations in the ground level $|g\rangle$ (blue line) and excited level $|e\rangle$ (green line) where the atoms are pumped by a resonant π pulse of duration $\Delta t = 0.001\mu s$, $0.1\mu s$, $1\mu s$, and $10\mu s$, respectively.

than $1/\Gamma$ the atom number transferred to the excited state reaches its maximum right after the pumping field is off as shown in Fig. S1 **a-c**. On the other hand, for $\Delta t > 1/\Gamma$, the spontaneous emission becomes important and significantly and suppresses the number of coherently excited atoms as depicted in Fig. S1 **d**. Nevertheless, even for $\Delta t = 10\mu s$ there are still around 10^5 atoms coherently excited. Therefore under the condition $\Omega_{pump} > \Gamma$ (i.e. $\Delta t < 1/\Gamma$), the π pulse pumping offers sufficient atom number for the superradiant process. The required π pulse laser power for

* scgou@cc.ncue.edu.tw
† wenteliao@cc.ncu.edu.tw

the relevant Lithium transition is estimated to be around $1-10\mathrm{W/cm^2}$ depending on Δt, and it is achievable with commercial laser systems.

Optical Bloch Equation. In the presence of counter-propagating coupling fields $\Omega_c^{\pm}$ and the superradiant fields $\Omega^{\pm}$, the dynamics of the EIT mirror is described by the optical-Bloch equation which reads [S1–S5]:

$$\partial_t\rho_{12} = -i\left(\Delta-\Delta_c\right)\rho_{12} - i(\Omega_c^+\rho_{13}^+ + \Omega_c^-\rho_{13}^-)/2 + i(\Omega^{+*}\rho_{32}^+ + \Omega^{-*}\rho_{32}^-)/2, \tag{S2}$$

$$\partial_t\rho_{13}^{\pm} = -\left(i\Delta+\Gamma/2\right)\rho_{13}^{\pm} - i\Omega_c^{\pm*}\rho_{12}/2 - i\Omega^{\pm*}\left(\rho_{11}-\rho_{33}\right)/2, \tag{S3}$$

$$\partial_t\rho_{23}^{\pm} = -\left(i\Delta_c+\Gamma/2\right)\rho_{23}^{\pm} - i\Omega^{\pm*}\rho_{21} + i\Omega_c^{\pm*}\left(\rho_{33}-\rho_{22}\right), \tag{S4}$$

$$\partial_t\rho_{11} = \Gamma\rho_{33}/2 + Im\left[\Omega^+\rho_{13}^+ + \Omega^-\rho_{13}^-\right], \tag{S5}$$

$$\partial_t\rho_{22} = \Gamma\rho_{33}/2 + Im\left[\Omega_c^+\rho_{23}^+ + \Omega_c^-\rho_{23}^-\right], \tag{S6}$$

$$\partial_t\rho_{33} = -\Gamma\rho_{33} - Im\left[\Omega^+\rho_{13}^+ + \Omega^-\rho_{13}^- + \Omega_c^+\rho_{23}^+ + \Omega_c^-\rho_{23}^-\right], \tag{S7}$$

where Γ is the spontaneous decay rate of the excited state $|3\rangle$ and ρ_{ij} is the element of the density matrix. In equations (S2)-(S7), all fast-oscillation exponential factors associating with center frequencies and wave factors have been eliminated, and only slowly-varying profiles are retained.

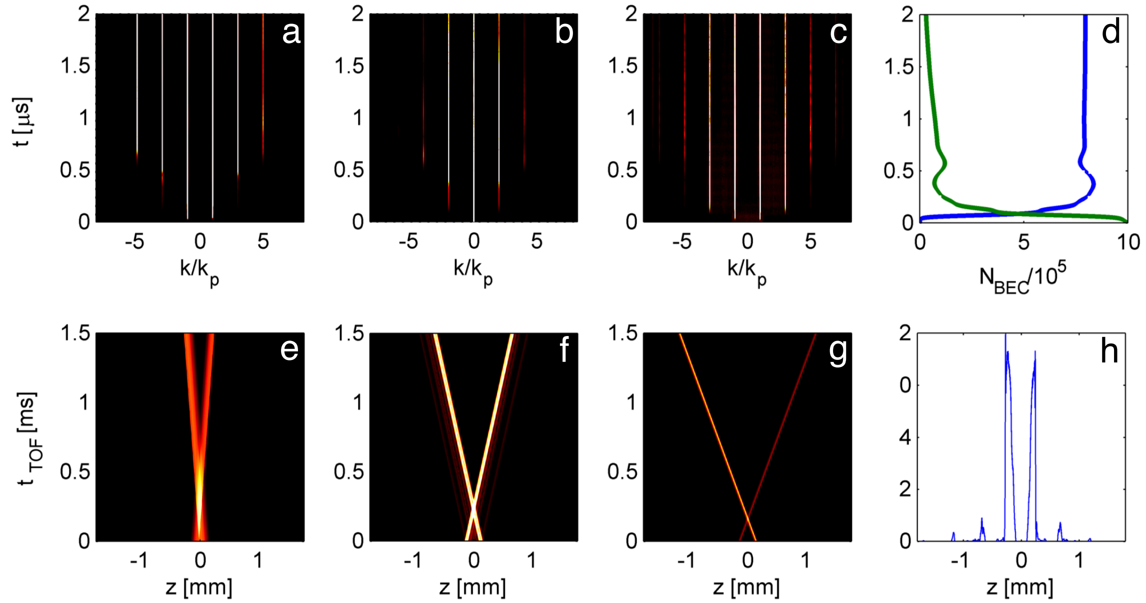

FIG. S2. Panels **a-d** show the dynamics of the binary BEC inside the EIT cavity of $d^{opt}=500$ and $\Omega_c=2\Gamma$ within 2 μs. The evolution of the condensate densities in momentum space are shown in panels **a** and **b** where the high odd k_p modes are generated in ψ_g while the even k_p modes are generated in ψ_e. Panel **c** shows the optical coherence σ_{eg}. The coherent transfer of the BEC particle number from states $|e\rangle$ (green line) to $|g\rangle$ (blue solid line) is shown. Panels **e-h** represent the time-of-fight simulation after the condensate is released from the cavity and harmonic trap. Panels **e-g** are the density components carry the momenta $\pm k_p$, $\pm 3k_p$, and $\pm 5k_p$, respectively. The total density profile $|\psi_g|^2$at $\mathrm{t_{TOF}}=1.8$ ms. The contrast of the 2D plots is adjusted for better visualization.

Dynamics of BEC. When the condensate is loaded in the dispersive cavity, in the presence of the interaction between the circulating SR fields and condensate, the condensate wave functions ψ_g and ψ_e constitute the superposition of discrete $\pm(2n+1)k_p$ and $\pm 2nk_p$ plane waves, respectively. In this manner the condensate wave function can be decomposed as :

$$\psi_g = \sum_{n=-\infty}^{\infty}\psi_g^{(n)}e^{i(2n+1)k_pz} \text{ and } \psi_\mathrm{e} = \sum_{\mathrm{n}=-\infty}^{\infty}\psi_\mathrm{e}^{(\mathrm{n})}\mathrm{e}^{\mathrm{i2nk_pz}},$$

where $\psi_{e,g}^{(n)}$ are slowly-varying profiles. As depicted in Fig. S2 **a** and **b**, the momentum-space density profile of single realization of $d^{opt} = 500$ and $\Omega_c = 2\Gamma$ shows the clear generation of these discrete $\pm nk_p$ modes. Furthermore the Fourier transform of the coherence σ_{eg} depicted in Fig. S2 **c** shows clear superposition of $\pm nk_p$ modes. The coherent SR-BEC interaction results in the Rabi oscillation in short time scale and transfers most of the atoms to the ground state while the rest of them decay to other states due to the incoherent spontaneous processes as shown in Fig. S2 **d**. The Rabi oscillation is the consequence of the strong atom-light coupling.

The generation of the highest $\pm nk_p$ mode is limited by the competition between the linewidth of the superradiance and the recoil-induced Doppler shift which is given by $\Delta\omega_n \approx \Gamma_{SR}$ where $\Delta\omega_n = \pm\hbar nk_p\omega/(mc)$ is the Doppler shift of n-th mode with ω the transition frequency and c the speed of light. In order to generate the harmonics as high as possible, one can use a heavier atom, e.g., ^{87}Rb, with lower recoil velocity.

After passing through the cavity, the dynamics of the condensate is described by the single component Gross-Pitaevskii equation which reads

$$i\hbar\partial_t\psi_g = \left(-\frac{\hbar^2}{2m}\partial_z^2 + \frac{m\omega_z^2 z^2}{2} + g_{gg}\left|\psi_g\right|^2\right)\psi_g, \tag{S8}$$

where we neglect the cross-species and atom-light interactions.

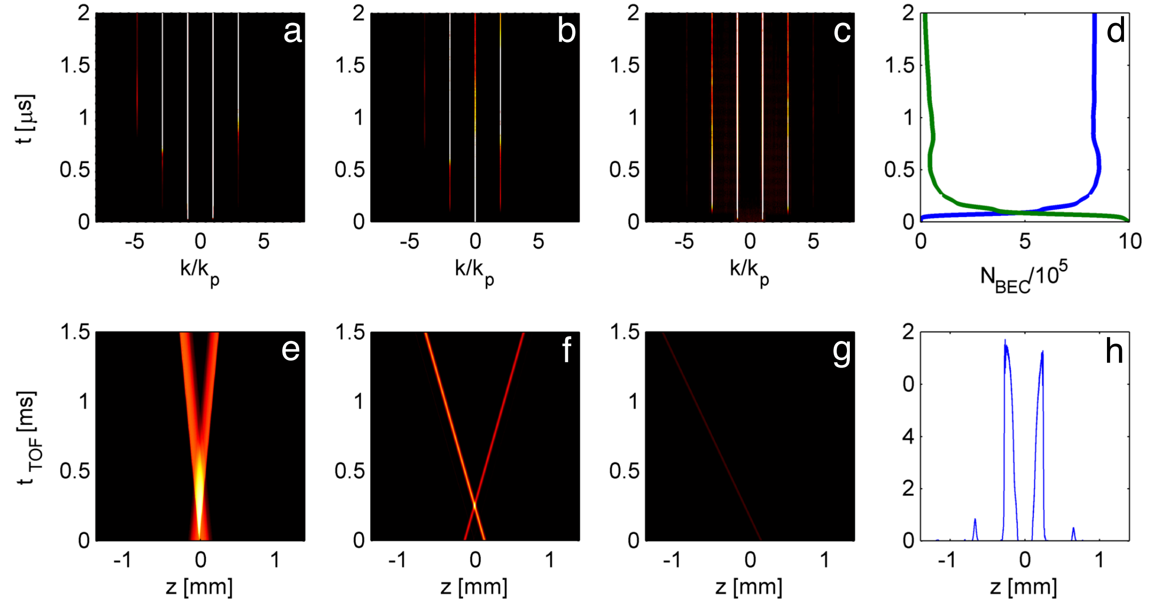

FIG. S3. Panels **a-d** The controllability of the cavity is shown for $d^{opt} = 500$ and $\Omega_c = 2\Gamma$ within 2 μs where the coupling fields are switched off at $t = 0.07$ μs. The evolution of the condensate densities in momentum space are shown in panels **a** and **b** where the only $\pm 3k_p$ modes are significantly generated in the ground state. Panel **c** shows the optical coherence σ_{eg}. The coherent transfer of the BEC particle number from states $|e\rangle$ (green line) to $|g\rangle$ (blue solid line) is shown. Panels **e-h** represent the time-of-fight simulation after the condensate is released from the cavity and harmonic trap. Panels **e-g** are the density components carry the momenta $\pm k_p$, $\pm 3k_p$, and $\pm 5k_p$, respectively. The total density profile $|\psi_g|^2$at $t_{TOF} = 1.5$ ms. The contrast of the 2D plots is adjusted for better visualization.

During the time of flight (TOF) measurement, the condensate would split into several atomic clouds which corresponds to different $\pm nk_p$ modes. To simulate the TOF dynamics, we follow the standard procedures in Refs. [S6–S8] to numerically integrated the time-dependent GPE, equation. S8. To describe the motion of the cloud with different $\pm nk_p$, the TOF simulation domain is extended to $7L$ where L is the simulation domain in the presence of harmonic trap.

As shown in Fig. S2 **e-h**, we perform the TOF simulation for $\Delta t_{TOF} = 1.5$ ms. In Fig. S2 **e-g**, the evolution of the condensed atoms carrying momenta are plotted and the velocities can be calculated from the slopes which agrees with the expected values, $\pm\hbar k_p/m$, $\pm 3\hbar k_p/m$, and $\pm\hbar k_p/m$. In Fig. S2 **h**, the total density distribution at $t_{TOF} = 1.5$ ms is shown where six density bumps that are symmetric to the origin can be observed. The two innermost density bump pair in Fig. S2 **h** corresponds to the $\pm k_p$ modes while the density bump pair located around $z = \pm 0.5$ mm is $\pm 3k_p$ modes and the outermost pair carries $\pm 5k_p$ momenta.

In addition, to control the generation of the momentum modes up to specific $\pm nk_p$ mode can be controlled by switching off the coupling fields Ω_c. For instance, in Fig. S3, the coupling fields are turned off at 0.07 μs where only $\pm 3k_p$ modes are significantly generated and the amplitude of Rabi oscillation is suppressed. In the TOF simulation as shown in Fig. S3 **e-h** only $\pm 3k_p$ modes can be clearly observed .

[S1] Marlan O. Scully and M. Suhail Zubairy, *Quantum optics* (Cambridge University Press, United Kingdom, 1997) (document) .

[S2] M Bajcsy, Alexander S Zibrov, and Mikhail D Lukin, "Stationary pulses of light in an atomic medium," Nature **426**, 638–641 (2003) .

[S3] Jin-Hui Wu, M. Artoni, and G. C. La Rocca, "Decay of stationary light pulses in ultracold atoms," Phys. Rev. A **81**, 033822 (2010) .

[S4] Yi-Hsin Chen, Meng-Jung Lee, Weilun Hung, Ying-Cheng Chen, Yong-Fan Chen, and Ite A. Yu, "Demonstration of the interaction between two stopped light pulses," Phys. Rev. Lett. **108**, 173603 (2012) .

[S5] Da-Wei Wang, Hai-Tao Zhou, Zhang Jun-Xiang Guo, Miao-Jun, Jörg Evers, and Zhu Shi-Yao, "Optical diode made from a moving photonic crystal," Phys. Rev. Lett. 110, 093901 (2013) (document) .

[S6] Lev Pitaevskii and Sandro Stringari, *Bose-Einstein Condensation* (Oxford University Press, Oxford, 2003) (document) .

[S7] T. Lahaye, J. Metz, B. Fröhlich, M. Meister, A. Gresmaier, T. Pfau, H. Saito, Y. Kawaguchi, and M. Ueda, "d-wave collapse and explosion of a dipolar bose-einstein condensate," Phys. Rev. Lett. **101**, 080401 (2008) .

[S8] E. Ruokokoski, J. A. M. Huhtamäki, and M. Möttönen, "Stationary states of trapped spin-orbit-coupled bose-einstein condensates," Phys. Rev. A **86**, 051607(R) (2012).
(document)
